# Nonylphenol Toxicity Evaluation and Discovery of Biomarkers in Rat Urine by a Metabolomics Strategy through HPLC-QTOF-MS

**DOI:** 10.3390/ijerph13050501

**Published:** 2016-05-14

**Authors:** Yan-Xin Zhang, Xin Yang, Pan Zou, Peng-Fei Du, Jing Wang, Fen Jin, Mao-Jun Jin, Yong-Xin She

**Affiliations:** 1 School of Chemistry and Chemical Engineering, Harbin Institute of Technology, Harbin 150090, China; yanxinzhangbj@163.com (Y.-X.Z.); zoupan0601@163.com (P.Z.); 2 Key Laboratory of Agro-Product Quality and Safety, Institute of Quality Standard & Testing Technology for Agro-Product, Chinese Academy of Agricultural Sciences, Beijing 100081, China; dupengfei2011@163.com (P.-F.D.); jinfenbj@163.com (F.J.); katonking@163.com (M.-J.J.); 0891syx@163.com (Y.-X.S.); 3 Key Laboratory of Agro-Product Safety and Quality, Ministry of Agriculture, Beijing 100081, China

**Keywords:** nonylphenol, metabolomics, exposure, HPLC-QTOF-MS, biomarker

## Abstract

Nonylphenol (NP) was quantified using liquid chromatography tandem mass spectrometry (LC-MS/MS) in the urine and plasma of rats treated with 0, 50, and 250 mg/kg/day of NP for four consecutive days. A urinary metabolomic strategy was originally implemented by high performance liquid chromatography time of flight mass spectrometry (HPLC-QTOF-MS) to explore the toxicological effects of NP and determine the overall alterations in the metabolite profiles so as to find potential biomarkers. It is essential to point out that from the observation, the metabolic data were clearly clustered and separated for the three groups. To further identify differentiated metabolites, multivariate analysis, including principal component analysis (PCA), orthogonal partial least-squares discriminant analysis (OPLS-DA), high-resolution MS/MS analysis, as well as searches of Metlin and Massbank databases, were conducted on a series of metabolites between the control and dose groups. Finally, five metabolites, including glycine, glycerophosphocholine, 5-hydroxytryptamine, malonaldehyde (showing an upward trend), and tryptophan (showing a downward trend), were identified as the potential urinary biomarkers of NP-induced toxicity. In order to validate the reliability of these potential biomarkers, an independent validation was performed by using the multiple reaction monitoring (MRM)-based targeted approach. The oxidative stress reflected by urinary 8-oxo-deoxyguanosine (8-oxodG) levels was elevated in individuals highly exposed to NP, supporting the hypothesis that mitochondrial dysfunction was a result of xenoestrogen accumulation. This study reveals a promising approach to find biomarkers to assist researchers in monitoring NP.

## 1. Introduction

Nonylphenol (NP) is an artificial environmental pollutant produced from alkylphenols and a scientifically well-known xenoestrogen and endocrine disruptor [1]. NP is ubiquitous in the environment, such as in the wastewater, drinking water, rivers, and animals. As important sources of NP, alkylphenol polyethoxylates (APEOs) are used all over the world and in only a few Asian countries [2,3,4]. According to a study in Taiwan, NP in the milk of women who often have fish oil is abundant, reaching a concentration of 4.47 μg/kg [5,6]. In a recent study of 62 drinking water samples in 31 major Chinese cities, NP was found in 55 samples with the median and highest concentrations of 27 and 558 ng/L, respectively. In all 62 water source samples there were median and maximum concentrations of 123 and 918 ng/L, respectively [7]. NP easily accumulates in animal and human bodies due to its hydrophobicity, demonstrating its great toxicity. Therefore, it is important to improve the accuracy of the evaluation of NP exposure and to monitor the health risk related to NP. 

Conventional toxicological studies showed that NP is a xenoestrogenic compound bound to estrogen receptors and competes for natural hormones [8]. NP at a certain concentration in the environment is highly toxic to many organisms. It affects the reproduction, endocrine, immune, and nervous system functions, as well as promoting tumor growth [9,10,11]. Sakazaki *et al.* [12] conducted a gene-based research to prove that NP could act on (ER)-α (ER: estrogen receptor) in the lymphocytes of mice. The reverse transcription-polymerase chain reaction (RT-PCR) results indicated that the expression of ER-α in the spleen cells of male and female mice obviously decreased, influencing the specific immune response of the organisms. Laurenzana [13] analyzed the effects of NP on the testosterone metabolism in the livers of male mice and on the expression of CYP450 using proteomics. The testosterone hydroxylase and 5α-reductase activities as well as the CYP3A and CYP2C protein levels are higher in male mice than in female mice. Genomics and proteomics are effective measures for studying the toxicity and mechanisms of toxicants [14]. However, some substances do not influence gene expression and protein synthesis. Genomics and proteomics are therefore not adequate for accurate toxicity evaluation of toxicants [15]. When toxicants interact with cells or organisms, the concentrations of the endogenous substances in the organisms during the key metabolic process will change. By applying this principle, researchers could measure metabolite changes to obtain global information about the binding sites, strengths, and action mechanisms of chemical toxicants [16].

Metabolomics applied to systems biology becomes popular and powerful in quantitatively measuring global changes in the individual metabolic profiles in response to biofluid analyses [17]. Recently, metabolomic approaches were used to analyze biofluid metabolic profiles and extensively employed to unravel the action mechanisms of toxicants in combination with multivariate data analysis [18,19]. However, there are few studies on the evaluation of NP toxicity using metabolomics. Sang Hee adopted GC-MS to measure the toxicological effects of NP based on rat urine [20]. Among the numerous analytical methods, liquid chromatography coupled with mass spectrometry (LC-MS), gas chromatography coupled with mass spectrometry (GC-MS), and nuclear magnetic resonance (NMR) have become major tools in metabonomics, with the advantages of high throughput and high resolution [21]. In contrast to GC-MS and NMR techniques, LC-MS was established as a more suitable choice for biomonitoring NP since it is superior in sensitivity, selectivity, and precision, even in low-concentration samples without derivatization. 

Based on these studies, an advanced strategy high performance liquid chromatography time of flight mass spectrometry (HPLC-QTOF-MS) was carried out to determine NP in the urine and plasma samples of ten rats in the control group, ten rats with low-dose exposure to NP, and ten rats with high-dose exposure to NP. HPLC-QTOF-MS was utilized in both positive and negative ion modes to detect endogenous metabolites in the urinary samples of rats exposed to NP. To investigate the global changes in the metabolic profiles after the rats were exposed to NP, a metabolomic approach was applied with multivariate data analysis (MVDA). The changes in the metabolite profiles with varying treatment amounts and time were analyzed, and a proper dose was chosen to further evaluate the toxic effect by principal component analysis (PCA). Orthogonal partial least-squares discriminant analysis (OPLS-DA) was then performed by SIMCA software (version 14.0, Umetrics, Umeå, Sweden) to identify discriminant variables. To find out all significant metabolites and define their correlations, the discriminating metabolites were selected with little loss. After the fragmental or isotope ions were selected from all discriminating metabolites, the database systems consisting of Metilin and Massbank were applied to metabolic correlation analysis. These database systems can identify the components of the discriminating metabolites that were used as potential biomarkers. Therefore, non-targeted and targeted metabolomic approaches may be employed as a tool for toxicology evaluation and biomarker identification. After glycine, glycerophosphocholine, 5-hydroxytryptamine, malonaldehyde, and tryptophan were found as biomarkers, the oxidative stress (determined by the 8-oxo-deoxyguanosine levels in urine) showed elevation in individuals highly exposed to NP, achieving the biomarker accuracy. 

## 2. Materials and Methods

### 2.1. Animal Treatment

Female Sprague–Dawley rats (190–220 g) were supplied by the Laboratory Animal Center of the Academy of Military Medical Sciences. They were fed with food and water available ad libitum in a temperature-controlled environment with a 12 h:12 h dark/light cycle applied. The female rats were randomly divided into three groups: control group (*n* = 10, labeled C1–C10), low-NP group (*n* = 10, labeled L1–L10), and high-NP group (*n* = 10, labeled H1–H10). NP was purchased from Tokyo Chemical Industry (purity 99%, Tokyo, Japan) and used as the standard. 4-NP was dissolved in corn oil and then separately given to the rats in a low dose (50 mg/kg/day) and a high dose (250 mg/kg/day) by gavage for 4 consecutive days, and corn oil of an equal volume without NP was gavaged into rats in the control group. The urine samples of the rats were collected with the metabolic cages during the following periods: 0 h (prior to gavage), first period (0–24 h), second period (24–48 h), third period (48–72 h), and fourth period (72–96 h). This research was conducted in accordance with the Declaration of Helsinki (2013), and all experimental protocols were approved by the Guidelines for Animal Experiments of the Chinese Academy of Agricultural Sciences (No. 20110320). 

### 2.2. Monitoring the NP Exposure Levels

#### 2.2.1. Sample Preparation

Extraction of NP from serum samples: 0.1 mL of 0.01 mol/L acetic acid-ammonium acetate buffer solution (pH 4.5) and 4.0 mL of n-hexane/ether (70:30 v/v) was added into a 0.50 mL serum sample. They were immediately stirred for 30 s and centrifuged at a speed of 4000 r/min for 5 min. Then, the liquid was divided into the upper organic layer (n-hexane/ether) and lower inorganic layers (acetic acid-ammonium acetate). The organic layer was collected into a bottle for evaporation in a water bath at 37 ± 1 °C. After the organic solvent was evaporated, 0.5 mL of acetonitrile was added to redissolve the non-volatilized components in the wall of the bottle. Then, the solution was determined by liquid chromatography tandem mass spectrometry (LC-MS/MS).

Extraction of NP from urine samples: 2 mL of 1 mol/L ammonium acetate solution (pH 5.0) and 10 µL of β-glucuronidase (20,000 U/mL) were added into 4 mL of urine samples [22], and the mixture was incubated at 37 °C overnight [23]. A Sep-Pak^®^ C18 solid-phase extraction (SPE) column was employed to clean each sample as follows: The column was preconditioned with 6 mL methanol–water (1:1). Then, 3 mL of the pretreated sample was loaded onto the SPE column. The column was air-dried and eluted with 3 mL methanol-water (3:7). Subsequently, the eluate was evaporated to dryness and redissolved in 200 µL methanol. This solution was filtered through a 0.22 mm membrane into an autosampler vial for HPLC-MS/MS detection.

#### 2.2.2. NP Analysis by HPLC-MS/MS

An Agilent 1200 series HPLC system (Agilent, Santa Clara, CA, USA) was used with a tandem mass spectrometer in ESI mode (API 5000, SCIEX, Middlesex, MA, USA) to analyze NP. Chromatographic separation was implemented with a Phenomenex C18 column (100 × 2 mm, 3 mm, Phenomenex, Torrance, CA, USA) and under 10 mmol/L ammonium acetate/methanol (1:9, v/v; mobile phase) isocratic conditions at a rate 0.2 mL/min.

NP analysis was performed using the following mass spectrometry parameters: precursor ions, 219.3; product ions, 133.2 and 147.3; curtain gas, 20 psi; voltage, −4500 V; ion source gas 1 and gas 2, both 40 psi; declustering potential, −30; collision energies, −40 and −38; entrance potential, −10; source temperature, 500 °C.

### 2.3. Metabolomics Analysis in Urine with HPLC-QTOF-MS

#### 2.3.1. Sample Preparation

Urine samples were stored at −80 °C before being analyzed by LC-MS/MS and HPLC-QTOF-MS. Prior to the analyses, the urine samples thawed at room temperature, and then were diluted with water until 1:3, centrifuged at 12,000 rpm for 10 min, and filtered through a 0.22 µm membrane. For the HPLC-MS/MS validation test, the urine samples containing the internal standards (chlorophenylalanine, 5 g/mL) were prepared. Finally, the 150 samples were bottled for measurement, and each sample was replicated 3 times.

The quality control (QC) samples were prepared by drawing 10 μL urine samples from the abovementioned 150 sample and mixing them up. To clearly assess chromatographic reproducibility, the first 5 consecutive QC samples in each batch were analyzed, and then 15 QC samples were repetitively analyzed by the analytical run following every ten urine samples. 

#### 2.3.2. HPLC-QTOF-MS Data Acquisition

A 1200 series HPLC (Agilent Technologies Inc., Santa Clara, CA, USA) was used in combination with a QSTAR^TM^ Elite QTOF-MS (AB SCIEX, Middlesex, MA, USA) for urine metabolic profile measurements. The urine analysis method that was established in our previous study was applied with other parameters detailed in the Supporting Information. Data acquisition was performed with Analyst QS 2.0 (AB SCIEX, Middlesex, MA, USA), and qualitative analysis was performed using PeakView 2.0 (AB SCIEX, Middlesex, MA, USA) equipped with Formula Finder, which was directly linked to the ChemSpider database (Royal Society of Chemistry, Cambridge, UK).

### 2.4. Processing and Statistical Analysis of MS Spectra Data

#### 2.4.1. Data Extraction and Normalization

MarkerView (version 1.2.1.1; AB SCIEX, Middlesex, MA, USA) was used for processing HPLC-QTOF-MS data, which involved data extraction, alignment, filtering, and normalization. Data extraction was performed using an automatic algorithm for finding the retention time (RT) peak; the RT range 0.5–60 min and m/z range 50–1200 were employed for metabolomic analysis. Then, the RT and m/z peaks were aligned with the tolerances of 0.2 min and 0.05 Da, respectively. Data matrices comprising molecular features depicted for each sample by (1) RT, (2) m/z value, (3) intensity, and (4) charge state (monoisotopic or isotopic), were automatically obtained by MarkerView, and the total area was normalized for each sample. 

#### 2.4.2. Multivariate Data Analysis

The mass spectral data in MarkerView were imported into the SIMCA software for multivariate statistical analysis. Principal component analysis (PCA) was performed in parallel with orthogonal partial least squares discriminant analysis (OPLS-DA) in Pareto mode. To investigate NP exposure, preliminary PCA was carried out for all three groups. OPLS-DA, a supervised pattern recognition approach, was applied for differentiation between the three groups. Ion-induced differences between the groups can be identified by *S*-plot in the OPLS-DA model. The model quality was given by two parameters, R2Y (goodness-of-fit), and Q2 (predictive ability). A permutation test (200 iterations) was conducted to validate each OPLS-DA model. The mass spectra with variable importance in projection (VIP) above 1 were selected in this study, and a non-parametric *t* test based on MarkerView with the critical *p*-value of 0.05 was used to further determine whether a significant difference existed between at least two groups for each metabolite obtained from the OPLS-DA models.

### 2.5. Identification of Metabolites

The following procedure was used in this study for metabolite identification: (1) the exact mass of the potential difference ion was used for predicting possible compositions; (2) the high-resolution mass spectra of precursor and product ions were used for determining the structure by comparison with the databases Metlin [24], Massbank [25], and HMDB [26]. Subsequently, commercially available standards were adopted to confirm the structure of some metabolites.

### 2.6. HPLC-MS/MS-Based Validation Test

The gradient conditions were obtained as follows: Solvents A and B were water with 0.1% formic acid and acetonitrile, respectively. The following multi–step elution gradient was used: 0–2 min, 95% solvent A; 2–15 min, 95%–30% solvent A, which was kept for 2 min; 18–23 min, 30%–5% solvent A, which was kept for 1 min and then changed back to the initial mobile phase rate and kept for 10 min. The flow rate of the mobile phases was 0.3 mL/min. The sample injection volume was 5 μL for all experiments. All of the potential biomarker candidates were detected by HPLC-MS/MS MRM mode. Levofloxacin and rhein as non-endogenous metabolites were selected as internal standards in positive ion mode and negative ion mode, respectively. The declustering potential (DP), collision energy (CE), and ion pairs were pre-optimized for each potential biomarker to give the best signal. The details were shown in Supplementary Table S1.

### 2.7. Quantitation of the Oxidative Stress Biomarker 8-oxo-deoxyguanosine (8-oxodG) in Urine

After being removed from the refrigerator (−80 °C), urine samples were thawed at room temperature and precipitated into protein on the tube by centrifugation at 12,000 r/min for 5 min. The levels of urinary-8-oxodG (U-8-oxodG) in the supernatant of urine were detected by the enzyme-linked immunosorbent assay (ELISA) kit obtained from Japan Institute for the Control of Aging (JaICA, Shizuoka, Japan). A standard curve was established for each ELISA plate within the concentration range of 0.5–200 ng/mL, and two parallel samples were prepared for each urine sample. The creatinine level in the urine sample was measured with the Jaffe method to calibrate the U-8-oxodG concentration. 

## 3. Results 

### 3.1. Determination of the Level of Exposure to Pesticides

The concentration of NP in plasma is usually recognized as an excellent indicator of NP exposure since it integrates all routes, pathways, and sources of exposure into biologically relevant measurements [27]. NP was measured according to the method described in Section 2.2.2, with the limit of detection (LOD) of 0.6 μg/L and relative standard deviation (RSD) of 2.6. 

**Figure 1:**
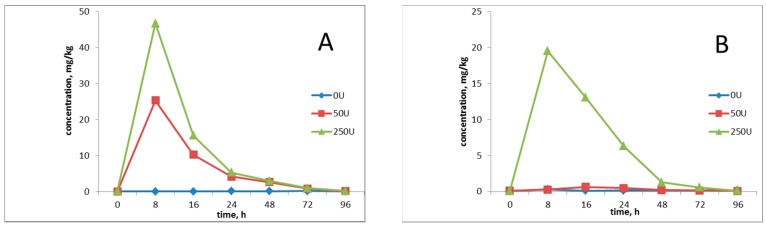
Distribution of NP in serum (**A**) and urine (**B**) in rats. NP concentrations below the limit of determination considered 0.

After NP was fed to rats by gavage, the distribution and clearance of NP in serum (A) and urine (B) changed with time (Figure 1). As shown in Figure 1A, NP was quickly absorbed after the rats were exposed to NP with different doses, and obvious absorption peaks (about 8 h later) appeared in the serum and then the concentrations gradually decreased. When the rats in the control group were exposed to the NP-free condition, no NP was detected in the serum. NP was maintained at a certain concentration in blood for a period, and then allowed to migrate to and accumulate in tissues, requiring subsequent experiments focusing on the study of NP-triggered variations of endogenous metabolites in the organisms. After the rats were exposed to NP at different doses, the excessive NP that failed to be absorbed was excreted in 48 h, as shown in Figure 1B. NP was almost not found in the urine samples of the control and low-dose group (50 mg/kg/day), whereas NP was detected in the urinary samples of the high-dose group (250 mg/kg/day), indicating that NP in the low-dose group was completely absorbed and that in the high-dose group was partially excreted, which was a result of metabolic saturation. The evaluation of the NP influence on organisms would be conducted in later experiments based on the 50 mg/kg/day dose group. 

### 3.2. Acquisition of Mass Spectrum Data

**Figure 2:**
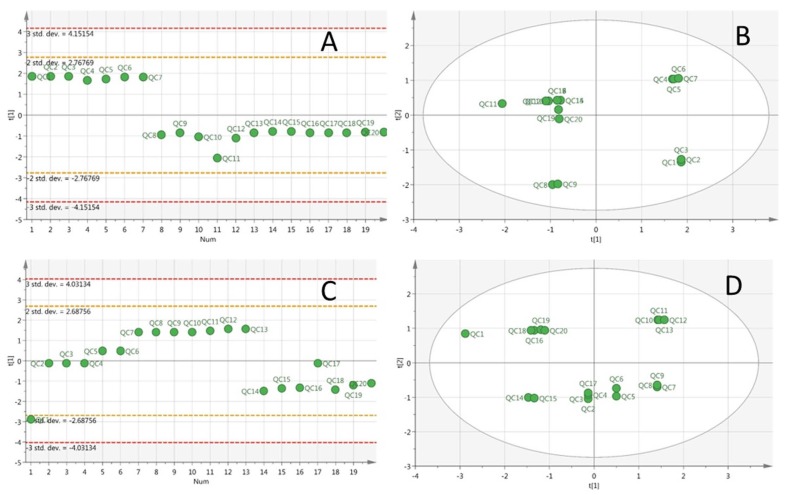
PCA of QC samples. Line plot (X-axis: run order; Y-axis: standard deviation) in positive ion mode (**A**); score plot (cycle represents 95% confidence interval) in positive ion mode (**B**); line plot in negative ion mode (**C**); and score plot in negative ion mode (**D**).

**Figure 3:**
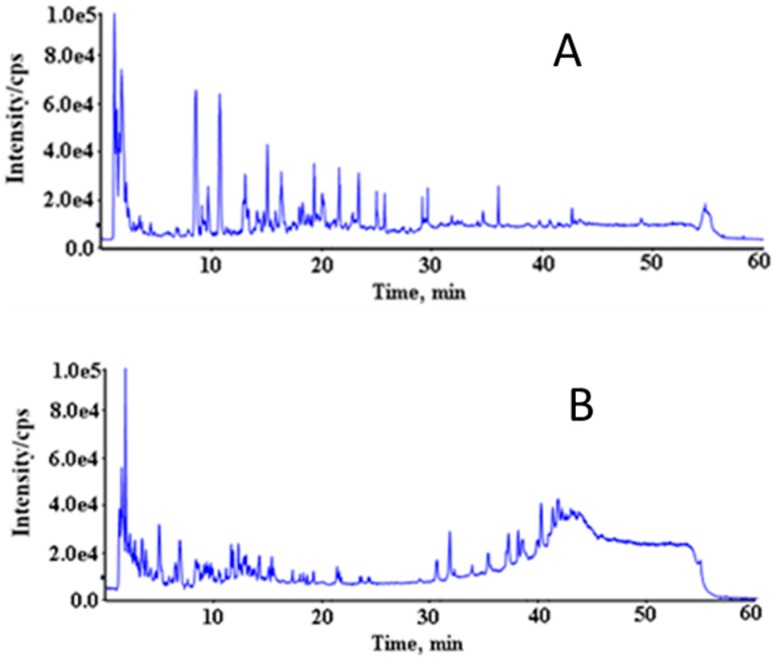
Typical chromatograms—total ion chromatogram (TIC) of a rat’s urine sample by LC-QTOF-MS, in positive ion mode (**A**) and negative ion mode (**B**).

A successful metabolomic study involves a high quality data set that produces a biochemical snapshot, with the endogenous small molecules or metabolites of an organism reflecting its temporal state [28]. In order to obtain reliable data, operation errors in sample preparation and instrumental errors must be minimized. In this study, samples from different groups were arranged in random order in each analysis batch. In addition, a QC sample was inserted between each of the 10 urine samples to monitor the instrument stability [29]. Principal component analysis (PCA) showed that the deviation variation of the QC samples were in the range of 2SD and all of the 20 QC samples were in the range of 95% confidence interval, indicating the data obtained from HPLC-QTOF-MS were valid (Figure 2). QC1 was out of the range of 2SD (Figure 2C), which may be caused by initial instability of the instrument. The above data quality evaluation showed that the significant differences obtained by PCA between different groups were a result of metabolite changes, but not experimental errors. The total ion chromatogram (TIC) of the urine samples analyzed with the optimized chromatography and mass spectrometry conditions was shown in Figure 3, which illustrated that chromatographic peaks were evenly distributed in the entire elution process. Analyst QS 2.0 showed that 1203 ions in positive ion mode and 486 ions in negative ion mode were detected. 

### 3.3. Dose Selection

MarkerView^TM^ 1.2.1 (AB SCIEX, Middlesex, MA, USA) was used to pretreat the collected mass spectrometric data, which were converted into an excel matrix with the mass-to-charge ratio being the variable after retention time calibration, peak extraction, peak alignment, and peak normalization. Then, the data were imported into the SMICA software. In our study, we always used PCA methods but because of the grouping and the characteristics of PCA, no obvious result was obtained. So, OPLS-DA was subsequently performed as a statistical model to find latent variables.

**Figure 4:**
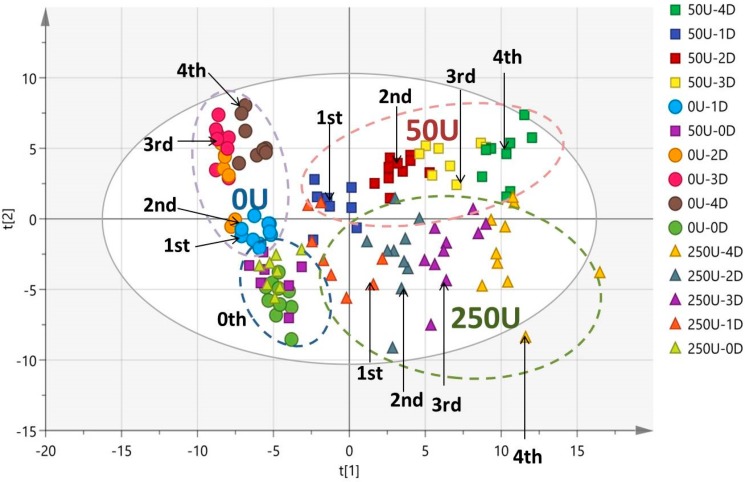
Score plot of OPLS-DA obtained based on the urine samples from the 0-unit group (green circle represents the period before exposure, blue circle represents 0–24 h, orange circle represents 24–48 h, red circle represents 48–72 h, and brown circle represents 72–96 h); 50-unit group (purple box represents the period before exposure, blue box represents 0–24h, red box represents 24–48 h, yellow box represents 48–72 h, and green box represents 72–96 h); and 250-unit group (green triangle represents the period before exposure, orange triangle represents 0–24 h, blue triangle represents 24–48 h, purple triangle represents 48–72 h, and yellow triangle represents 72–96 h).

**Figure 5:**
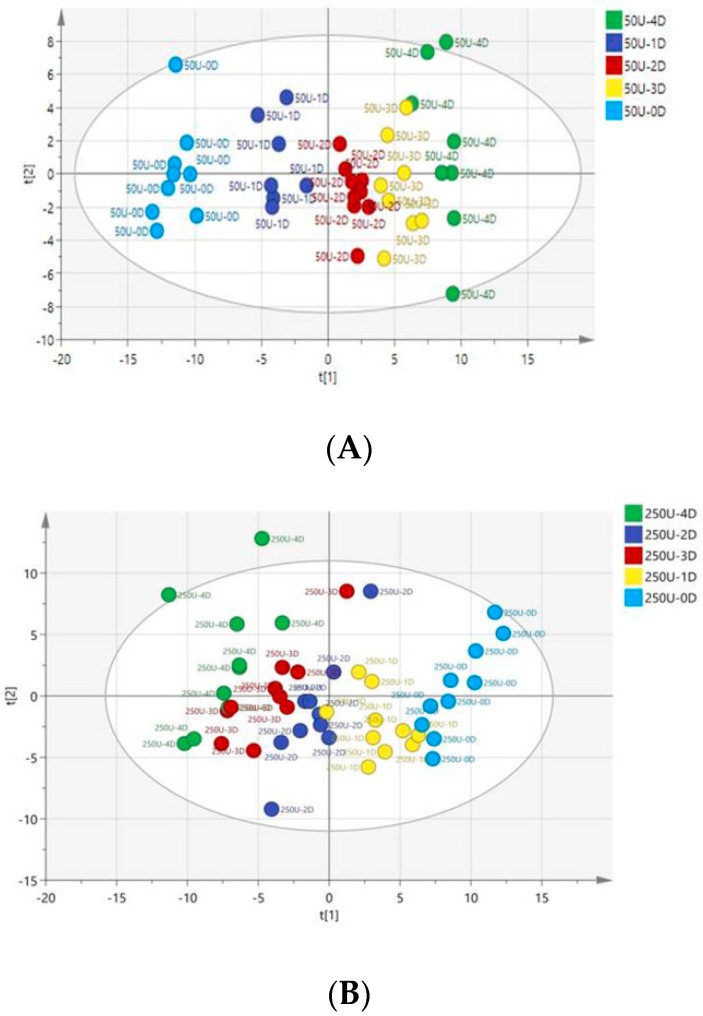
Score plot of OPLS-DA obtained based on the urine samples from the 50-unit group (**A**) and 250-unit group (**B**).

OPLS-DA with *R*2*X* (0.296), *R*2*Y* (0.795), and *Q*2 (0.832) was obtained in the score plot (Figure 4). As shown in Figure 4, the data points of the 0-unit class were not separated from each other. However, we could clearly discriminate the data points of the 50- and 250-unit classes, which started at the same point on day 0. After they were exposed to NP from the first day, the data points of these groups were gradually scattered and distributed in different regions, which showed serial patterns with the following treatment time from day 1 to day 4. Our analysis results of the patterns indicated that the metabolic profiling in rat urine could facilitate the study of the NP’s toxicological effects. After metabolic patterns were visually inspected with the values of *R*2*Y* and *Q*2 considered, we found that distinct clusters were categorized into the normal control, 50 units, and 250 units (Figure 4). The pattern of the 50-unit class was more greatly differentiated from the normal control (0 unit) than the experimental group (250 units) (Figure 5). The 50-unit values (*R2Y* = 0.836, *Q2* = 0.878) had higher coefficients than the 250-unit values (*R2Y* = 0.641, *Q2* = 0.659). Therefore, we speculated that the pattern of the 50-unit NP treatment offered the most distinguishable pattern for the evaluation of the NP effect on endocrine metabolism. 

### 3.4. Multivariate Data Analysis of HPLC-TOF-MS Spectra

**Figure 6:**
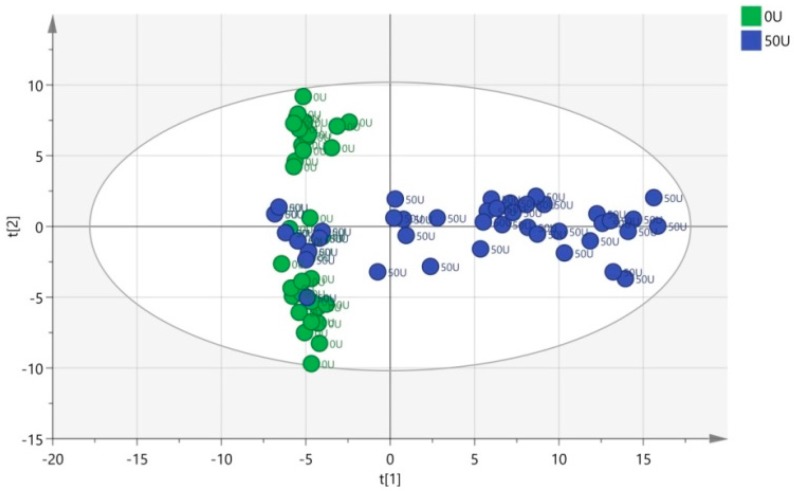
Score plot of principal component analysis (PCA) obtained based on the urine samples from the 0-unit group (green circle) and 50-unit group (blue circle).

A PCA score plot was drawn for the mass spectrometric data of both the 0-unit group (green circle) and 50-unit group (blue circle) (Figure 6). *R2Y* and *Q2* of the PCA model are respectively 0.797 and 0.684, indicating that the PCA model has excellent goodness-of-fit and predictability and is suitable for subsequent data analysis. A clear separation suggesting that the disturbance of urinary metabolites was significantly different between the exposure group and the control group. In Figure 6, several samples (green circles) in the 0-unit group and samples (blue circles) in the 50-unit group coincided in a region. This is because they were both collected on day 0, revealing that urine before NP exposure contained approximately the same metabolites.

**Figure 7:**
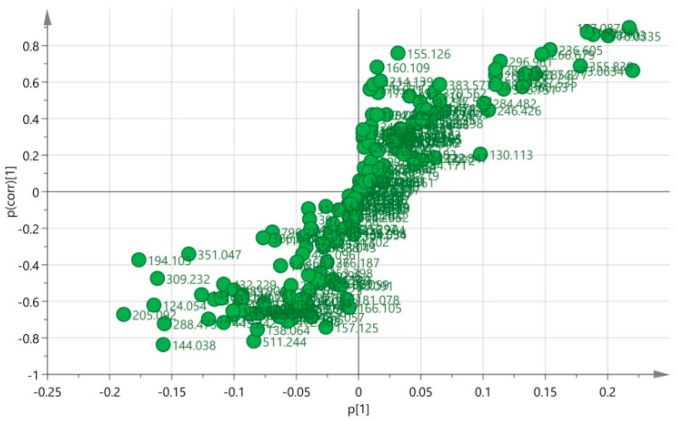
The loading plot (*S*-plot) of OPLS-DA for the 0 unit *vs.* 50 units.

The OPLS-DA model was established to discover NP-initiated metabolic alterations and revised to use multivariate data to differentiate the 0- and 50-unit group. The loading plot (*S*-plot) of OPLS-DA in Figure 7 showed that all variables represented an *S* symbol and a few variables at the two ends of the *S* symbol were scattered, which led to the discrimination of the two groups. Therefore, the best biomarkers were located in the two corners on the upper right and lower left. The molecular structures of the variables were identified, and potential biomarkers might be found for the toxicants.

**Figure 8:**
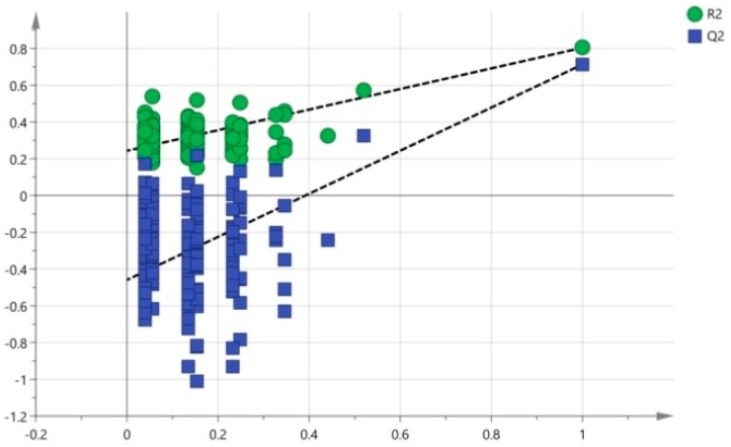
The 200-time permutation tests for the PCA and OPLS-DA models.

The model (*R2X* = 0.286, *R2Y* = 0.783, and *Q2* = 0.702) was highlighted as an excellent high-quality model, which properly classified all samples. The results demonstrated that 28.6% of the variables (*R2X*) could be used to explain 78.3% of the variations between the exposure group and the control group (*R2Y*). The results of projection validity indicated that the average prediction capability (*Q2*) was 70.2%. The difference between *R2Y* and *Q2* was less than 0.2 and the *Q2* value was greater than 50%, revealing an excellent predictive capability [30]. A 200-time permutation test was conducted to further validate the established model. The goodness-of-fit (*R2*) and predictive capability (*Q2*) of the unmodified model were then demonstrated (Figure 8). The acquired *R2Y* (green circle) and *R2Y* of the actual model formed a regression line with an intercept of 0.115, which was theoretically required to be less than or equal to 0.3. The *Q2* (blue box) and *Q2* of the actual model formed a regression line with an intercept of −0.705, which was required to be no greater than 0.05 in theory. The obtained values met the critical value requirements. The validity test results indicated that the mass spectrometric data matrix obtained from batches of urine samples had established a reliable identification model after the above treatment, which could be used for later differential variable acquisition. 

The correlations between variables and components were determined, as shown in Figure S1. In the OPLS-DA model, there were more than 48 variables considered important (VIP > 1) (VIP means variable importance in projection values), among which 20 variables were considerably different between the 0- and 50-unit group (*t* test; *p* < 0.05). The ions and their *p*-values were given in Table S1. 

The ions with VIP values greater than 1 and their *p*-values were listed in Table S1. The changing trend of these ions with the *p*-values less than 0.05, which showed a difference between the 50-unit group and the 0-unit group, were also presented. The most significant changes were observed at the bottom of the *S*-plot with a downward trend for the 50-unit group and on the upper part with an upward trend for the control group. The trend could also be observed in Figure S2. The m/z value 205.092 (up-regulated for the dose group compared with the control, Figure S2a) and m/z value 177.087 (down-regulated for the dose group compared with the control, Figure S2b) were used as an example. 

### 3.5. Biomarkers Identification 

**Figure 9:**
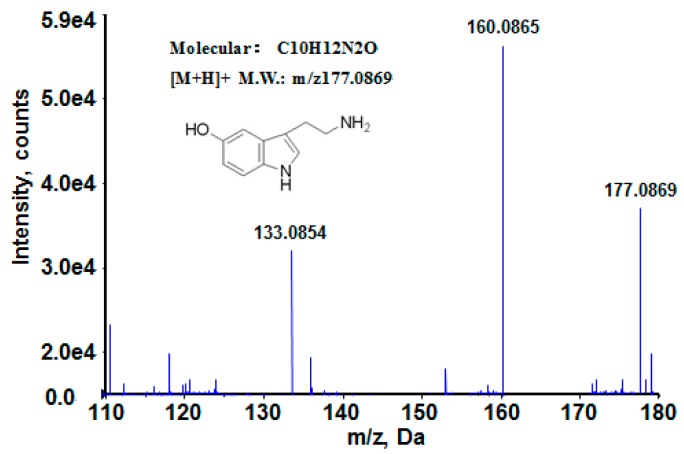
The MS spectrum and product ion spectrum of the metabolite ion at m/z 177.0869 obtained by TOF-MS/MS.

The metabolite identification procedure was described using 5-hydroxy-tryptamine as an example. The structure of 5-hydroxy-tryptamine is 3-(2-aminoethyl)-5-oxyindole (Figure 9), which has the retention time of 12.6 min in mass spectrometry, where the [M + H]^+^ molecular ion was at m/z 177.0869. Figure 9 shows the high-resolution LC-MS/MS spectrum of the metabolite, which indicates that the isotopic peak of the metabolite [M + H]^+^ ion is extremely low, implying S is not included in the compound. According to the preceding conditions and accurate mass value of the molecular ion, the molecular formula in Analyst QS 2.0 was used to calculate possible compositions, and the only possible element obtained was C_10_H_13_N_2_O^+^, by which the metabolite was inferred as 5-hydroxy-tryptamine. The product ions in the MS/MS spectrum could all be reasonably elucidated. The m/z 160.0865 was produced from the [M + H]^+^ ion by discarding an NH_3_ molecule, while the m/z 133.0854 was generated from the [M + H]^+^ ion with the loss of 44.0025 Da, which should result in CH_2_CH_2_NH_2_. This is because 5-hydroxy-tryptamine is easily broken to form a stable 5-membered ring. After the comparison with 5-hydroxy-tryptamine standards in the MS/MS spectra, the possible biomarker’s structure was finally determined as 5-hydroxy-tryptamine. This method was conducted to identify other metabolites with a similar flow. 

### 3.6. Potential Biomarkers Validation by Targeted Metabolomics Based on HPLC-MS/MS

**Figure 10:**
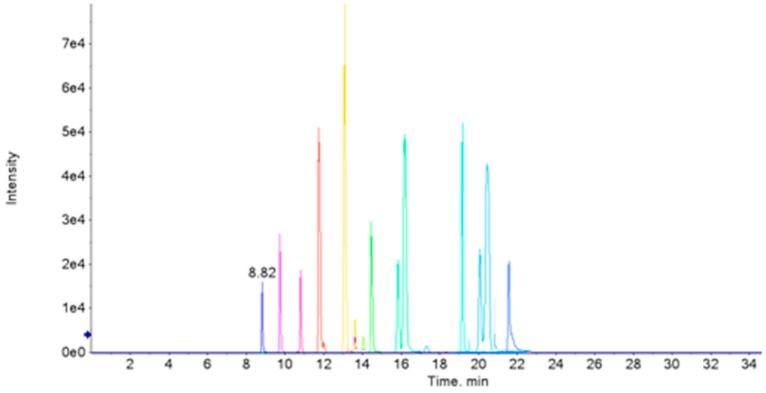
The typical XICs of the HPLC-MS/MS MRM mode analysis in potential biomarkers.

By PCA and *t* test, 20 ions that introduced the inter-group differences were selected. To further validate the reliability of potential biomarkers, a targeted metabolomic experiment based on HPLC-MS/MS was conducted. 0.1 g levofloxacin and rhein were selected as the internal standards to monitor the stability, and RSD of peak area in each sample was less than 7% and 8%, respectively. The typical XICs of the 20 biomarker candidates in HPLC-MS/MS in MRM mode are shown in Figure 10. According to the results of independent *t* test (*p* < 0.05) between the experimental and control groups, 12 metabolites were screened out of the previous 20 biomarker candidates. The 12 metabolites were selected after the validation test and regarded as the more reliable potential biomarkers. Finally, according to the methods mentioned in 3.5, five metabolites were identified by structural analysis and comparison with the standards, and the structures and metabolic pathways of the biomarkers were determined, as shown in Table 1.

### 3.7. Association of NP Exposure and Oxidative Stress

The U-8-oxodG content can be used to reflect the DNA oxidative damage degree of an organism, which is a new type of internationally recognized sensitive index and biomarker for evaluating oxidative damages and oxidative stress states of organisms.[31] To define the relationship of NP exposure with the potential of oxidative stress, U-8-oxodG was measured. The U-8-oxodG concentrations were 1.247 ± 0.018 (nmol/mmol creatinine ± standard error of the mean (SEM)) in the control group and 5.932 ± 0.021 in the 50-unit group. A significant (*p* < 0.05, *t* test) increase was discovered in the U-8-oxodG concentration (mean ± SD) in the individuals. The result suggested a positive correlation between U-8-oxodG and NP and indicated that the mitochondrial dysfunction and reactive oxygen species (ROS) may increase, which is consistent with our hypothesis. 

## 4. Discussion

The test results showed that the tryptophan level in the urine of rats exposed to NP was extremely low. When there was inflammation, the tryptophan was converted into kynurenine by inflammatory lymphatic tissues [32,33]. It is expected that the exhaustion of tryptophan may be caused by the inflammation after contact with NP [34]. An important study has elucidated the process of inflammatory intestinal disorder, in which male mice with ACE2 genes knocked out were used [35]. ACE2 could activate the expression of the tryptophan transporter in intestinal epithelial cells. Therefore, the mice in the test seriously lacked tryptophan. When exposed to dextran sulfate sodium (DSS), the mice had more obvious responses to the occurrence of colitis compared with the mice in the control group. This enhanced the filtration of inflammatory cells. A series of further experiments indicated that the mice in the control group had similar responses after being fed with feedstuff in which tryptophan was greatly lacking. Therefore, NP affects the synthesis of tryptophan in organisms and may lead to the most severe inflammatory intestinal diseases [36]. The disturbance of this metabolic pathway revealed that NP may damage the immune system. 

A large number of reports indicated that NP had an influence on the anti-oxidative balance of cells and therefore triggered oxidative stress [37]. The increase in the malondialdehyde concentration and decrease in the glutathione level implied the increase of oxyradical generation [38]. Glycine is a type of protective agent, which clears ROS and suppresses inflammatory responses. Earlier research data indicated that exposure to organophosphorus and pyrethroid insecticides may trigger the generation of ROS [39]. We suspected that the increase of tryptophan in urine may provide protection against oxidative stress initiated by exposure to NP. GPC had a critical function in the structural integrity of cell membrane [40]. The GPC increase indicates the start of the protection mechanism in cell damage, which might be caused by oxidative stress. 

Using tryptophan as its source, 5-HT was synthesized and catalyzed into 5-hydroxyindoleacetic acid (5-HIAA) by monoamine oxidase (MAO) before being excreted with urine. Normally, 5-HT was not contained in urine but the 5-HIAA metabolite might exist. This study demonstrated that the nonylphenol-contaminated urine of rats had an apparently increased 5-HT concentration [41]. It was reported that NP entering the kidney tissues of rats might produce or trigger to produce a large amount of ROS by interacting with some cells, which would lead to interruption of the antioxidant defense system and consequent damage to the rats’ kidneys [42]. From the test results of this study, it can be inferred that NP may cause damage to the rats’ kidney tissues, especially kidney tubules [43].

**Figure 11:**
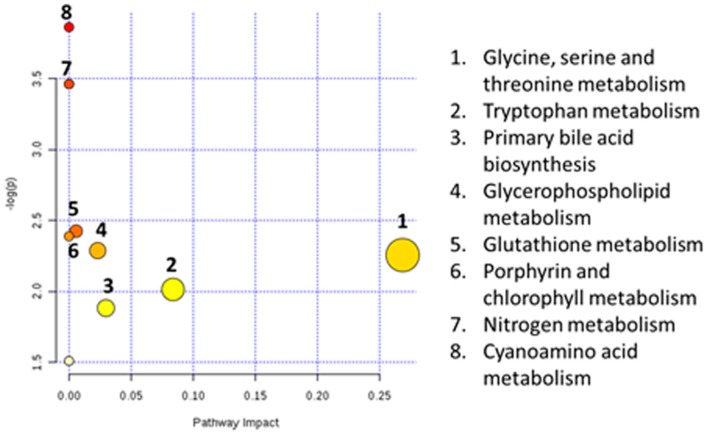
The metabolic pathways affected by NP exposure.

According to an investigation, the structure of NP is similar to 17β-estradiol (E2), and NP can compete to inhibit the combination of E2 with the estrogen receptor (ER) of the target organ [44,45]. It has been reported that E2 could increase the 5-HT level in the nervous system by inhibiting the MAO activity [8,46]. The MAO dysfunction is considered to be associated with nervous system diseases [47]. In this study, NP was considered as a type of environmental estrogen and simulated the function of E2 to suppress the MAO activity in the rats’ bodies so that 5-HT accumulated to a high level due to the interruption of decomposition and metabolism. As a result, the nervous system of the organism was injured. The metabolic pathways affected by NP exposure are shown in Figure 11. MetaboAnalyst was used to analyze the metabolic pathways affected by NP exposure. In Figure 11, the term “log (P)” on the Y axis is the transformation of the original *p* value calculated from the enrichment analysis; the term “Impact” on the X axis is the pathway impact value calculated from the pathway topology analysis. The bubble area is proportional to the impact of each pathway, with different colors denoting the significance from the highest in red to the lowest in white.

Many kinds of substances in the environment lead to the oxidative stress in animal or human bodies. In this experiment, we did not detect other exogenous environmental pollutants except nonylphenol, because the exposure study was carried out by feeding nonylphenol standards in metabolic cages without other pollutants. Even if the environment contained other pollutants, their contents were considered to be very low, with little impact on the oxidative stress in rats. This could be achieved only in animal experiments, in which the experimental conditions were well controlled. However, animal-to-human extrapolation still remains a challenge because human beings are affected not only by pollutant exposure, but also by many kinds of environmental pollutants with similar structures. To be specific, there is always a possibility that other chemicals apart from NP may contribute to oxidative stress through a synergic mechanism [48].

## 5. Conclusions

This study is the first exploratory work aiming at the discovery of the link between metabolic changes and NP exposure in rats. 

The metabolic spectrum analysis based on HPLC-TOF-MS was applied to the metabolites in the urine of rats exposed to NP in this study. PCA was used to analyze the obtained data, and the score plot of PCA indicated an obvious urinary metabolite change in the exposure and control groups. Five metabolites were acquired by accurate mass value match, multiple database search, and standard sample comparison. They were glycine, glycerophosphocholine, 5-hydroxytryptamine, malonaldehyde, and tryptophan. The biological functions of the biomarkers indicate that NP can cause multiple toxic effects on humans such as oxidative stress, mitochondrial dysfunction, nervous system damage, and immune system damage. Furthermore, it was helpful to experimentally confirm the oxidative stress, which supports our assumption. This method was established based on NP contamination, and was a product of the systematic study on the effects of NP exposure by using new techniques. It was extremely important for optimizing NP toxicity identification and evaluating toxicity risks. 

## Figures and Tables

**Table 1:** LC-MS/MS data of the discriminant metabolites related to NP exposure and their identification results.

Metabolite	Molecular Formula	VIP Score	*p*-Value	Monitored Structure	Monitored M.W.	Exact Mass	Trend
5-hydroxytryptamine	C_10_H_12_N_2_O	2.59281	0.036	M + H	177.0869	176.0950	upward
tryptophan	C_11_H_12_N_2_O_2_	2.39113	0.027	M + H	205.0918	204.0899	downward
glycine	C_2_H_5_NO_2_	2.40796	0.041	M + H	76.0335	75.0320	upward
glycerophosphocholine	C_8_H_20_NO_6_P	2.2673	0.032	M + H	258.1031	257.1028	upward
malonaldehyde	C_3_H_4_O_2_	2.89664	0.023	M + H	73.0634	72.0211	upward

M.W.: expressed molecular weight.

## Supplementary Materials

The following are available online at www.mdpi.com/1660-4601/13/5/501/s1, Figure S1: VIP distribution in the OPLS-DA model, Figure S2: (A) The m/z value 205.092 upward for the 50 units group compared with the 0 unit) and (B) the m/z value 177.087 (downward for the 50 units group compared with the 0 unit), Table S1: The conditions of HPLC-MS/MS to validate potential biomarkers, Table S2: The VIP, *p*-value, and trends of different ions found by OPLS-DA.

## Abbreviations

NP	Nonylphenol
ESI	Electrospray ionization
MS	Mass spectrometry
Q-TOF MS	Quadrupole time-of-flight mass spectrometer
HPLC	Ultra high performance liquid chromatography
IDA	information-dependent acquisition
MVDA	multivariate data analysis
PCA	principal component analysis
OPLS-DA	orthogonal partial least-squares discrimination analysis
8-oxodG	8-oxo-deoxyguanosine
