# Supplementary material for: Nonylphenol Toxicity Evaluation and Discovery of Biomarkers in Rat Urine by a Metabolomics Strategy through HPLC-QTOF-MS
Source: Int J Environ Res Public Health. 2016 May 14;13(5):501. doi: 10.3390/ijerph13050501 (PMC4881126; doi:10.3390/ijerph13050501)
Supplement: Supplementary file 1 [file ijerph-13-00501-s001.pdf]

# Supplementary Materials: Nonylphenol Toxicity Evaluation and Discovery of Biomarkers in Rat Urine by a Metabolomics Strategy through HPLC-QTOFMS

**Yan-Xin Zhang, Xin Yang, Pan Zou, Peng-Fei Du, Jing Wang, Fen Jin, Mao-Jun Jin and Yong-Xin She**

## 2. Materials and Methods

### *2.3. Metabolomics Analysis in Urine with HPLC-QTOF-MS*

#### 2.3.2. HPLC-QTOF-MS Data Acquisition

The HPLC system was equipped with a Waters XBridge™ C18 column (2.1 × 150 mm, 5 μm), and the column temperature was set to 25 °C. The mobile phases for metabolic fingerprinting consisted of 0.1% formic acid in Milli-Q water and 5 mM ammonium acetate in Milli-Q water (solvent A, positive electrospray ionization (ESI+) and negative electrospray ionization (ESI–), respectively), acetonitrile (solvent B), and methanol (solvent C) in both (ESI+) and (ESI–) analyses. The following multi-step elution gradient was used: 0–2 min, 90% solvent A; 2–40 min, 90%–5% solvent A, which was kept for 10 min; 50–51 min, 5%–90% solvent A, which was kept for 10 min and then changed back to the initial mobile phase rate; 40–50 min, 30% solvent B; 0% solvent B in other periods. The flow rate of the mobile phases was 0.3 mL/min. The sample injection volume was 5 μL for all experiments.

The ion source was a separated ESI ion source in TurboSpray™. In ESI+ mode, the initial parameters for metabolomics were as follows: ion spray voltage, 5500 V; nebulizing gas pressure (GS1), 60 psi; drying gas pressure (GS2), 50 psi; ion source temperature, 500 °C; focusing potential, 265 V; curtain gas pressure, 25 psi; declustering potential, 80 V. In ESI-mode, the ion spray voltage was −4200 V; the declustering potential was −60 V; the focusing potential was −265 V; the other parameter settings were the same with ESI+. At the same time, the TOF-MS and information-dependent acquisition (IDA) methods were used to collect MS and MS/MS spectra. The methods involved a TOF-MS experiment with spectra ranging from m/z 50 to 1200 for metabolomic analysis. Dynamic background ions were subtracted to acquire MS spectra, which were recorded with automatic collision energy. In this way, low- and high-energy fragment ions were both present in a single spectrum.

### *2.6. HPLC-MS/MS-Based Validation Test*

**Table S1.** The conditions of HPLC-MS/MS to validate potential biomarkers.

| Q1 Mass (Da) | Q3 Mass (Da) | DP (V) | CE (V) | Ion Mode |
|---|---|---|---|---|
| 194.1 | 125/68.5 | 80 | 25/40 | + |
| 73.2 | 52.1/30.3 | 60 | 20/30 | + |
| 177.1 | 130/85.2 | 65 | 25/40 | + |
| 76.0 | 45.9/29.2 | 50 | 25/30 | + |
| 205.1 | 145.1/103.8 | 60 | 30/35 | + |
| 258.2 | 180.2/95.4 | 60 | 30/40 | + |
| 160 | 108/75.2 | 70 | 15/25 | + |
| 285.4 | 139/104.1 | 60 | 20/25 | + |
| 141.1 | 85.2/67 | 50 | 20/25 | + |
| 246.4 | 156/88.3 | 60 | 30/35 | + |
| 247.5 | 156.2/102 | 70 | 30/40 | + |
| 432.2 | 312/204.8 | 80 | 30/35 | + |
| 296.9 | 157.3/89 | 70 | 25/40 | + |
| 283.2 | 122.3/90.3 | –65 | –35/–30 | − |

| | | | | |
|---|---|---|---|---|
| 445.2 | 203.8/134.7 | −70 | −25/−30 | − |
| 119.1 | 76/43.2 | −70 | −22/−28 | − |
| 268.7 | 172.2/126 | −60 | −30/−40 | − |
| 111.0 | 66/45.2 | −65 | −35/−40 | − |
| 216.9 | 156.3/89 | −70 | −35/−30 | − |
| 498.3 | 232.8/165.9 | −60 | −30/−40 | − |

## 3. Results

### *3.4. Multivariate Data Analysis of HPLC-TOF-MS Spectra*

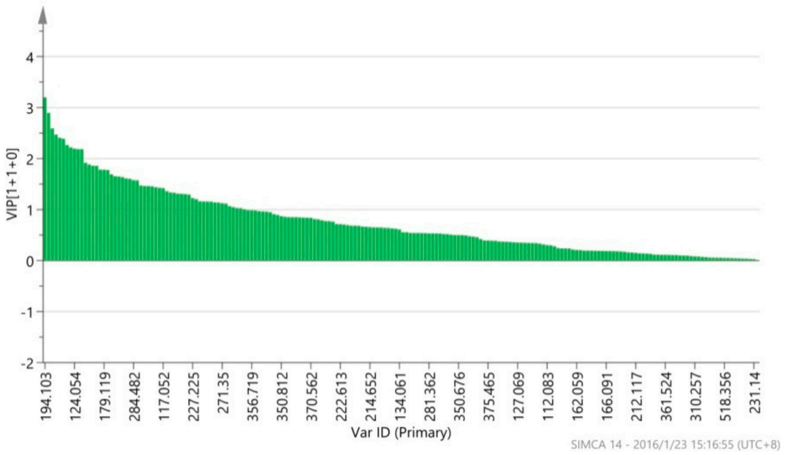


**Figure S1.** VIP distribution in the OPLS-DA model.

**Table S2.** The VIP, *p*-value, and trends of different ions found by OPLS-DA.

| Varable ID (Primary) | M2 VIP (1 + 0 + 0) | *p*-Value | Trends |
|---|---|---|---|
| 194.103 | 3.20173 | 0.032 | upward |
| 73.0634 | 2.89664 | 0.046 | upward |
| 177.087 | 2.59281 | 0.021 | upward |
| 309.232 | 2.47136 | 0.148 | downward |
| 76.0335 | 2.40796 | 0.022 | upward |
| 205.092 | 2.39113 | 0.046 | downward |
| 258.103 | 2.2673 | 0.027 | upward |
| 355.839 | 2.22234 | 0.147 | downward |
| 124.054 | 2.19655 | 0.074 | upward |
| 351.047 | 2.187 | 0.059 | upward |
| 160.041 | 2.18602 | 0.038 | downward |
| 144.038 | 1.91818 | 0.064 | upward |
| 288.473 | 1.88484 | 0.086 | downward |
| 236.605 | 1.86005 | 0.541 | downward |
| 130.113 | 1.85904 | 0.356 | not obvious |
| 796.756 | 1.78462 | 0.086 | not obvious |
| 179.119 | 1.78306 | 0.126 | upward |
| 266.679 | 1.77678 | 0.054 | upward |
| 285.377 | 1.69169 | 0.026 | not obvious |
| 141.13 | 1.65387 | 0.017 | upward |
| 246.426 | 1.64913 | 0.039 | not obvious |
| 360.631 | 1.63763 | 0.076 | upward |
| 247.535 | 1.61167 | 0.025 | upward |

| | | | |
|---|---|---|---|
| 301.742 | 1.6044 | 0.082 | upward |
| 284.482 | 1.57816 | 0.137 | not obvious |
| 125.099 | 1.57493 | 0.167 | upward |
| 399.577 | 1.47335 | 0.095 | upward |
| 432.229 | 1.4631 | 0.022 | not obvious |
| 306.731 | 1.46207 | 0.052 | not obvious |
| 211.972 | 1.42949 | 0.564 | not obvious |
| 117.052 | 1.42164 | 0.078 | upward |
| 296.961 | 1.36267 | 0.016 | upward |
| 283.192 | 1.33699 | 0.046 | not obvious |
| 445.242 | 1.33155 | 0.037 | upward |
| 119.081 | 1.31319 | 0.009 | not obvious |
| 268.686 | 1.31028 | 0.019 | upward |
| 284.302 | 1.30409 | 0.057 | not obvious |
| 122.07 | 1.29508 | 0.127 | not obvious |
| 227.225 | 1.22513 | 0.146 | not obvious |
| 116.087 | 1.20611 | 0.166 | upward |
| 111.078 | 1.16245 | 0.039 | upward |
| 314.246 | 1.1605 | 0.057 | not obvious |
| 216.917 | 1.15817 | 0.044 | not obvious |
| 366.265 | 1.15671 | 0.176 | not obvious |
| 240.168 | 1.11843 | 0.178 | not obvious |
| 498.326 | 1.06967 | 0.038 | not obvious |

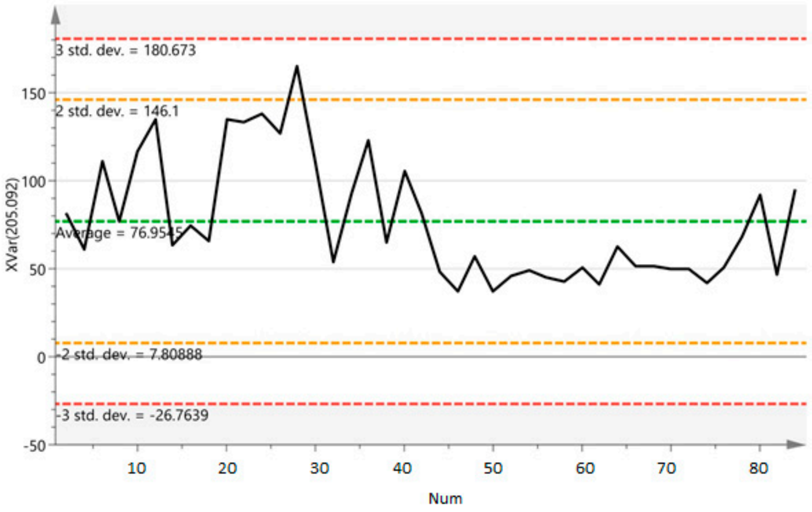


(**A**)

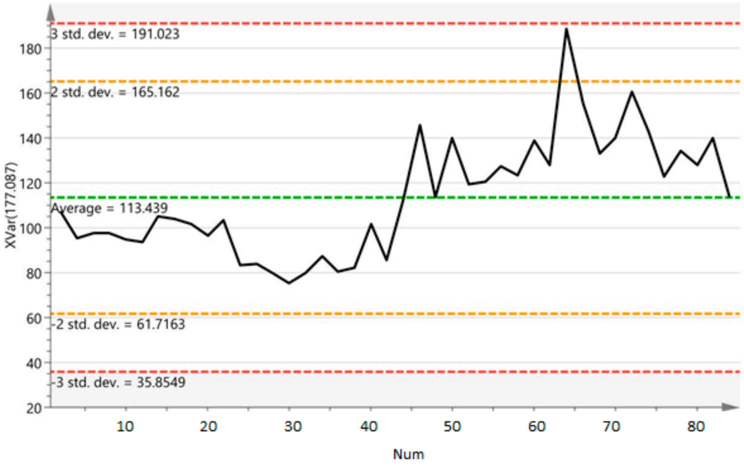


(**B**)

**Figure S2.** (**A**) The m/z value 205.092 (upward for the 50 units group compared with the 0 unit) and (**B**) the m/z value 177.087 (downward for the 50 units group compared with the 0 unit).

© 2016 by the authors; licensee MDPI, Basel, Switzerland. This article is an open access article distributed under the terms and conditions of the Creative Commons by Attribution (CC-BY) license (http://creativecommons.org/licenses/by/4.0/).
